# Submental flap for vascularized lymph node transfer; a CTA‐based study on lymph node distribution

**DOI:** 10.1002/jso.26117

**Published:** 2020-07-15

**Authors:** Vera A. A. Paulus, Harm Winters, Stefan Hummelink, Sascha Schulten, Dietmar J. O. Ulrich, Dalibor Vasilic

**Affiliations:** ^1^ Department of Plastic Surgery Radboud University Medical Center Nijmegen The Netherlands; ^2^ Department of Plastic Surgery Erasmus Medical Center Rotterdam The Netherlands

**Keywords:** lymphedema, submental lymph nodes, upper extremity, VLNT

## Abstract

**Background:**

Amongst various options of vascularized lymph node transfers, the submental flap has the lowest risk for iatrogenic lymphedema. The aim of this study was to gain insight into distribution, number, and size of lymph nodes along the mandible using computed tomography angiography (CTA).

**Methods:**

A total of 52 CTA scans of head/neck region were evaluated retrospectively. Lymph nodes in the submental and submandibular region, related to the origin of the submental artery, were recorded using a three‐dimensional coordinate system, and standardized using an iterative closest point algorithm. Results were analyzed for gender, location, size, and number.

**Results:**

The mean number and size of lymph nodes were 5.30 ± 2.00 and 5.28 ± 1.29 mm, respectively. The mean distance of the lymph nodes to the origin of the submental artery was 25.53 ± 15.27 mm. There was no significant difference between both sides when comparing size (left: 5.39 ± 1.28; right: 5.17 ± 1.34; *P* = .19), number (left: 5.46 ± 2.10; right: 5.17 ± 1.96; *P* = .49), and distance (left: 24.78 ± 12.23; right: 26.32 ± 14.73; *P* = .19). No significance was found between males and females concerning number (*P* = .60), size (*P* = .50), and distance (*P* = .06).

**Conclusion:**

The variance of lymph node distribution along the mandible may warrant conducting a CTA scan to maximize the number of transferred lymph nodes and aid in flap design.

**Abbreviations**
CTA: computed tomography angiography
ICP: iterative closest point
LVA: lymphaticovenous anastomosis
VLNT: vascularized lymph node transfer

## 1 INTRODUCTION

Lymphedema is a chronic and progressive swelling due to lymphatic dysfunction and occurs mostly in extremities. The initial treatment of lymphedema consists of compressive decongestive therapy that utilizes special compression garment, manual lymph drainage, and skin care.^1^, ^2^, ^3^ Where conservative therapy focuses on preventing further swelling of the extremity, surgical options can be considered as an attempt to slow down and halt the pathophysiological deterioration process associated with chronic lymphedema.

In lymphaticovenous anastomosis (LVA), lymph fluid is diverted into the venous system.^2^, ^3^, ^4^ An important limitation of an LVA is that it can only be performed in the early stages of lymphedema, as a superficial functional lymphatic vessel is necessary for a successful procedure and outcome.^2^, ^3^

Patients who are lacking suitable functional lymphatic vessels, necessary for an LVA, can be candidates for a vascularized lymph node transfer (VLNT). In this procedure, lymph nodes are transferred from a healthy donor site to the affected limb. VLNT aims to tackle the physiologic impairment by replacing the lost lymph nodes and potentially induce the regeneration of lymphatic vessels. To date, various VLNT donor sites have been described such as the groin flap, the supraclavicular flap, the submental flap, the gastroepiploic flap, the mesenteric and the thoracic lymph node flap.^2^, ^5^, ^6^, ^7^, ^8^, ^9^, ^10^, ^11^, ^12^ With this arsenal of free lymph node flaps available, the preferred flap for treating lymphedema of the extremity is the submental flap, due to a large number of lymph nodes and low risk for iatrogenic lymphedema.^2^, ^13^, ^14^, ^15^

The improvement of lymphedema after the surgery is correlated to the number of lymph nodes transferred and their vascularization, hence imparting a meticulous flap design and harvest.^15^, ^16^ The imperative of including the maximal number of the lymph nodes with perinodal tissue is traded off against the increasing size of the flap, which, in turn, results in a larger visible submental scar. The submental lymph nodes, however, may all be located proximal to the origin of the submental artery, making the distal part of the flap skin redundant, hence departing from the rationale calling for a larger flap. It would, therefore, be beneficial to gain insight into the distribution of the lymph nodes in this area to determine optimal balance between inclusion of the most lymph nodes to be transferred and size of the flap.

The aim of this study was to investigate the distribution, number, and size of lymph nodes along the mandible using computed tomography angiography (CTA) to gain insight into the variability of the lymph nodes in this area.

## 2 MATERIALS AND METHODS

In this retrospective study, patients who had undergone a head/neck CTA for a follow‐up of a head and neck malignancy, or for staging of tumors located in other regions than head and neck, were included in the study. Head/neck scans were acquired in a helical mode in craniocaudal direction at 120 kV tube voltage where Iomeron 300 mg/mL (Bracco Imaging SpA, Milan, Italy) had been used as an intravenous contrast agent. Exclusion criteria for this radiologic study comprised of lymphadenopathy or lymph node dissection in the CTA report, insufficient image quality, presence of tumor, or metastatic disease or unclear origin of the submental artery.

In total 52 CTA scans (n = 23 male, n = 29 female) of satisfactory imaging quality were included to assess 100 sides (n = 48 bilateral, n = 4 unilateral) for lymph node anatomical features. The mean age of the group was 61.77 ± 13.78 years. The mean body mass index was 26.68 ± 4.86 kg/m^2^.

### 2.1 Analysis of CTA scans

**Figure 1:**
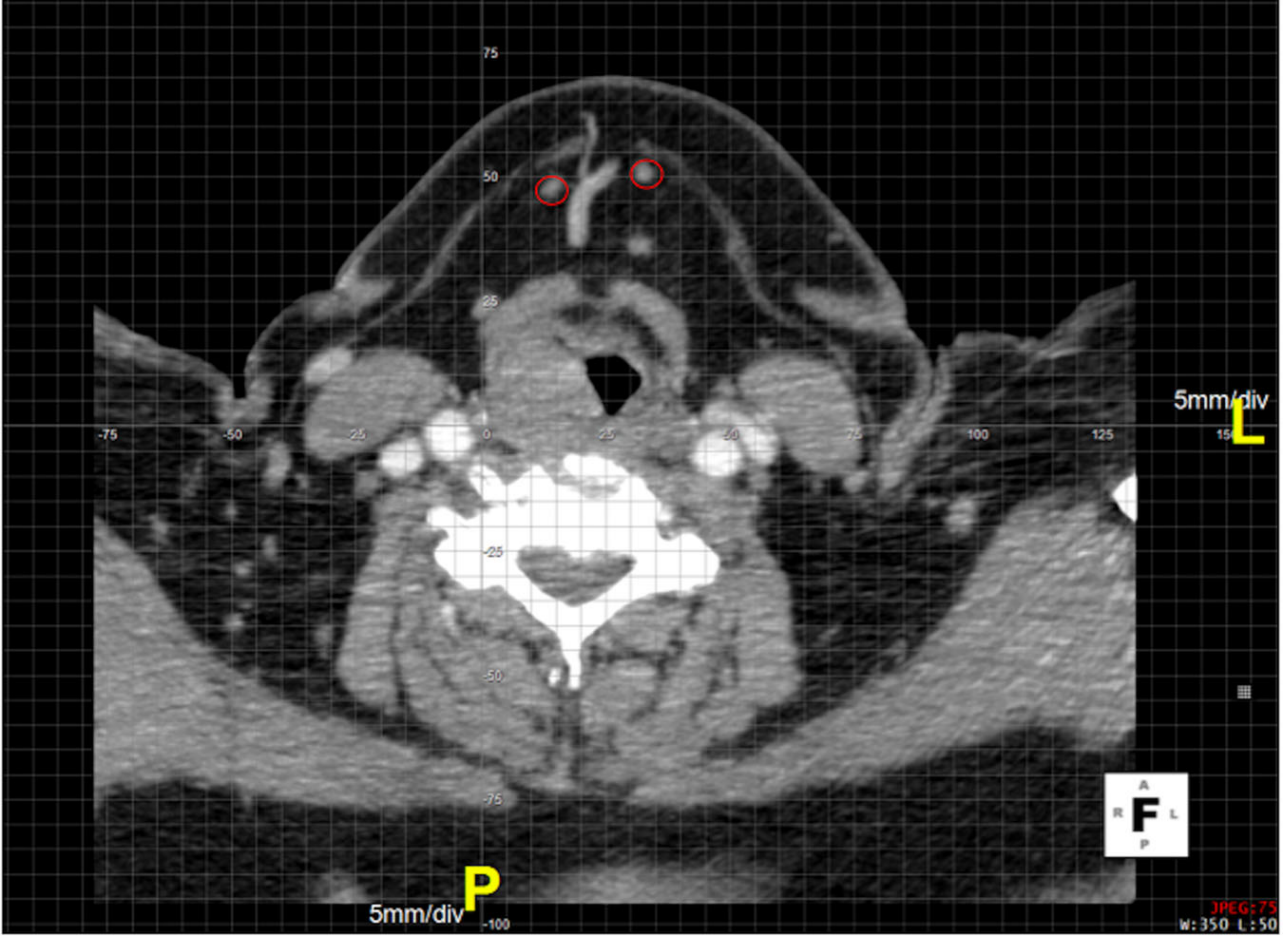
Axial slice of a head/neck CTA scan. The origin of the right submental artery is centered in the coordinate system. Two lymph nodes encircled in red. CTA, computed tomography angiography

The submental artery branches of the facial artery and provides the essential blood supply to the submental flap. During the procedure of harvesting the submental flap, its origin from the facial artery acts as an anatomical landmark. Therefore, lymph nodes were recorded at each side using the origin of the submental artery as a central coordinate in the CTA scans (Figure 1). Lymph nodes in the submental and submandibular region (IA and IB)^17^ were recorded between the hyoid bone, the angles of the mandible, the mental protuberance, and the bellies of the digastric muscle. In each evaluated side the coordinates of the landmarks were determined; the ipsilateral and contralateral angle of the mandible and the chin. This process was performed on all included CTA scans using post‐processing software Aquarius iNtuition Viewer 4.4.12 (TeraRecon, Foster City, CA).

The locations of lymph nodes were recorded by a first observer (VP); validation was performed by randomly selecting 10% of the recorded scans and re‐evaluated by a second observer (SH). We compared the number of nodes at the levels IA and IB strictly confined within predefined anatomical landmarks (hyoid bone, angles of the mandible, mental protuberance, and belly of the anterior digastric muscle). An observed agreement of 0.90 was calculated during this validation process.

The gathered data were analyzed using MATLAB 2016b (The MathWorks Inc, Natick, MA). The heterogeneous lymph node coordinates were standardized through an iterative closest point (ICP) algorithm, unifying all lymph node coordinates into a single coordinate system. The mean coordinates of the ipsi and contralateral angle of the mandible and the chin were calculated from the entire dataset and used as fixed registration points for the ICP algorithm. To obtain a rotation and translation matrix, the moving registration points included landmarks pertaining to each individual patient. All lymph node coordinates from the same patient could be transposed into a single coordinate system using the individually obtained rotation and translation matrix.

Three‐dimensional graphic presentation of all standardized lymph nodes was achieved by creating spheres at each individual location with the corresponding radius of the lymph node within MATLAB. The spheres representing lymph nodes, mandible, and blood vessels were rendered within AutoDesk MeshLab (San Rafael, CA), retaining global anatomical orientation.

The Students *t* test (Excel 2013, Office, Microsoft) was used for subgroup analysis, and parameters were considered significant at the value *P* ≤ .05.

## 3 RESULTS

A total of 530 lymph nodes were located in 100 evaluated one‐sided level IA/IB cervical regions. The mean number and size of lymph nodes were 5.30 ± 2.00 and 5.28 ± 1.29 mm, respectively. The mean distance from the lymph nodes to the origin of the submental artery was 25.53 ± 15.27 mm (Table 1). In the analysis of the number of lymph nodes between either side (left: 5.46 ± 2.10; right: 5.17 ± 1.96) no significant difference was found (*P* = .49). Furthermore, the size of the lymph nodes (left: 5.39 ± 1.28 mm; right: 5.17 ± 1.34 mm) and distance to the origin of submental artery (left: 24.78 ± 12.23 mm right: 26.32 ± 14.73 mm) showed no significant difference (*P* = .43 and *P* = .19, respectively).

**Table 1:** Patient characteristics of study population

Included scans	52/154
Excluded scans	102/154
Gender	
Male	29 (55.77%)
Female	23 (44.23%)
Included sides on scans	100 (50 left, 50 right)
Mean age	61.77 ± 13.78 y
Mean body mass index	26.68 ± 4.86 kg/m^2^
Mean amount of lymph nodes	5.30 ± 2.00 (n = 530)
Mean size of lymph nodes	5.28 ± 1.29 mm
Mean distance of lymph nodes to origin submental artery	25.53 ± 15.27 mm

**Figure 2:**
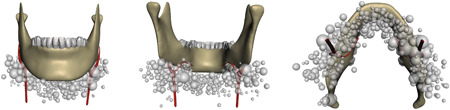
Distribution and size of lymph nodes. Gray spheres correspond with size and location along a standardized mandible relative to the submental artery (red). A, anterior‐posterior view. B, posterior‐anterior view. C, caudal‐cranial view

**Figure 3:**
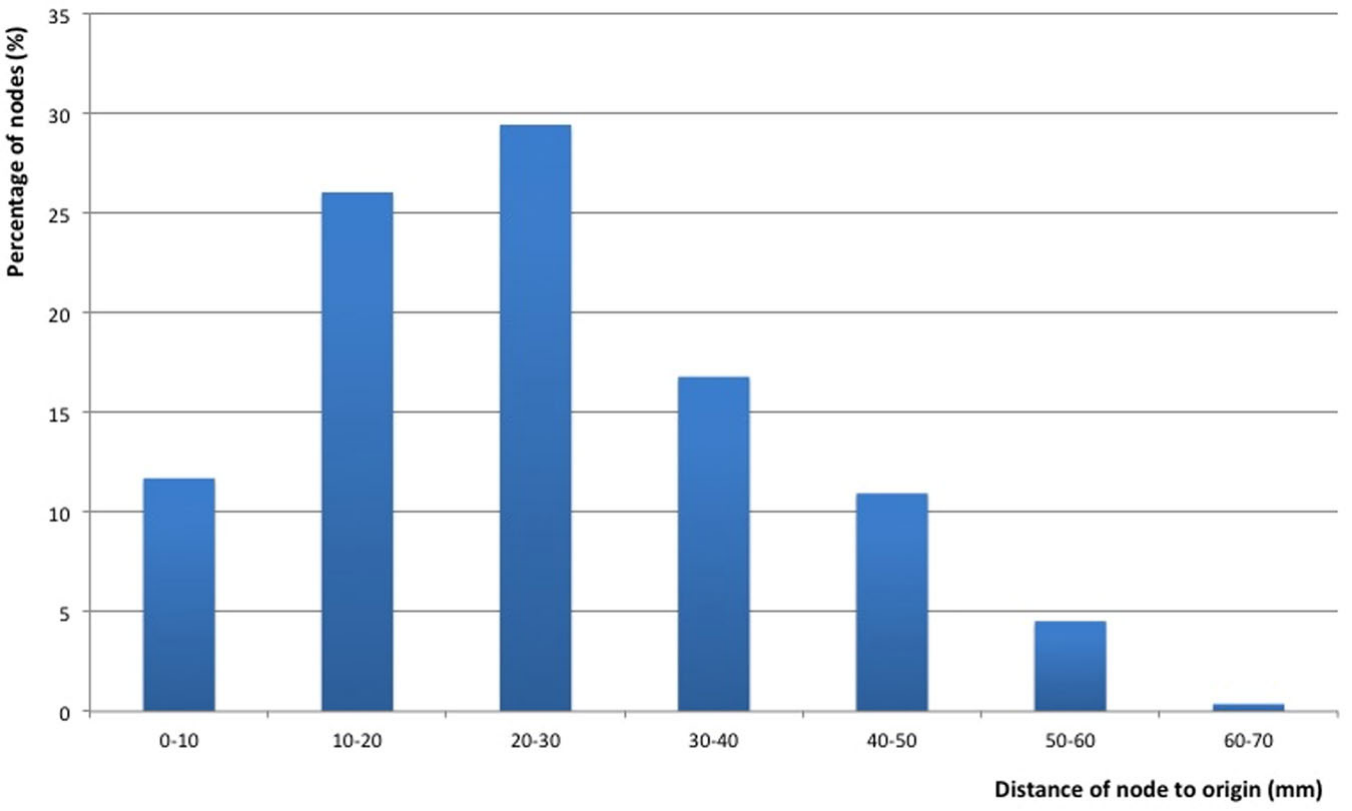
Percentage of lymph nodes in relation to the distance to origin of the submental artery

When comparing the number of lymph nodes between males and females (male: 5.20 ± 2.05; female: 5.41 ± 1.96), no significant difference was found (*P* = .60). The size of lymph nodes did not significantly differ either (male: 5.20 ± 1.13 mm, female: 5.38 ± 1.46 mm, *P* = .50), nor did the distance to the origin of submental artery (male: 26.56 ± 13.32 mm, female: 24.37 ± 13.68 mm, *P* = .06). These results are summarized in Table 2. Figure 2 depicts the distribution of lymph nodes along the mandible. The majority of the lymph nodes were approximately found between 1 and 4 cm from the origin of the submental artery as illustrated in Figure 3.

**Table 2:** Number, size, and location of lymph nodes, comparison between side and gender

	Left	Right	*P* value	Male	Female	*P* value
No. of lymph nodes	5.46 ± 2.10	5.17 ± 1.96	.49	5.20 ± 2.05	5.41 ± 1.96	.60
Size, mm	5.39 ± 1.28	5.17 ± 1.34	.43	5.20 ± 1.13	5.38 ± 1.46	.50
Distance to origin, mm	24.78 ± 12.23	26.32 ± 14.73	.19	26.56 ± 13.32	24.37 ± 13.68	.06

When comparing the number of lymph nodes between left and right side for each patient, 18.8% (n = 9) no significant difference was found in our study population. Most patients (50%, n = 24), showed a difference of one lymph node between either side. Albeit much less frequent, a difference of two nodes (20.8%, n = 10), three nodes (8.3%, n = 4), and four nodes (2.1%, n = 1) between the cervical sides was also found (Table 3).

**Table 3:** Difference in amount of nodes between both sides of the neck per individual patient

Difference in number of lymph nodes between both sides	Number of patients (%)
0	9 (18.8)
1	24 (50)
2	10 (20.8)
3	4 (8.3)
4	1 (2.1)

## 4 DISCUSSION

VLNTs have gained a momentum in treatment of early and end‐stage lymphedema.^18^ Following the VLNT, the lymphedema accumulates in the flap and its lymph nodes, followed by redistribution of the collected fluid into the bloodstream.^19^ Although the exact working mechanism remains unknown, couple of studies, both in animals and humans, showed promising results.^2^, ^3^

Various VLNT donor sites have been described such as the groin flap, the supraclavicular flap, the submental flap, the gastroepiploic flap, the mesenteric and the thoracic lymph node flap.^2^, ^5^, ^6^, ^7^, ^8^, ^9^, ^10^, ^11^, ^12^ Compared to the other flaps the submental flap has a relatively high number of lymph nodes and a low risk for iatrogenic lymphedema.^2^, ^13^ While some studies looked into the anatomy and lymph node characteristics of the submental region based on cadaveric dissections,^3^, ^20^ others have focused on the clinical setting describing the value and limitations of ultrasound and MR for visualization of submental lymph nodes.^13^, ^21^ To our knowledge no previous study described the added value of a preoperative CTA modality pertaining to the exact number and anatomical localization of level IA/B lymph nodes to maximize the number of vascularized lymph nodes to be transferred within the smallest size of the submental flap devisable.

The findings of this study show that anatomical location of the submental lymph nodes varies, both among patients as well as between the left and right side of the mandible. CTA scan can be of value for the surgeon to identify the cervical side with the most lymph nodes available in level IA/IB and based on these findings create the most suitable design for the flap. Furthermore, knowledge of the location of the nodes helps to preserve not only the hilar blood supply of the nodes, but also the surrounding fatty tissue by keeping a safe distance to the nodes while dissecting the submental flap. This perinodal fat contains high levels of growth factor (VEGF‐C), which plays an important role in subsequent lymphatic regeneration, and should be preserved.^1^, ^18^, ^20^

To preserve a maximal number of the submental nodes the soft tissue around the submental and facial artery bifurcation is included in the flap.^3^

An average number of 5.3 nodes was found in the submandibular triangle, disagreeing with previously published results (3.2 and 3.3 lymph nodes, respectively, found in a cadaver study of Tan and Cheng).^3^, ^20^ This indicates that possibly more lymph nodes can be transferred when an accurate assessment of a CTA scan is done preoperatively. Most nodes were found between approximately 1 and 4 cm distance from the origin of the submental artery. This indicates that a surgeon can safely navigate to the origin of the submental artery by first locating the facial artery, resting assured that lymph nodes will not be missed or denuded during the dissection. The immediate vicinity of the submandibular gland itself is the most demanding area of the surgical dissection because of the imperative to preserve level IB lymph nodes, their vascularity, and vascularity of the flap given the anatomical variability of the submental artery. There is a great variability of the anatomy pertaining to submental artery course at the superior border of the submandibular gland, but also traversing inside the gland and between its lobes. Level IB lymph nodes are intimately juxtapositioned to the submandibular gland and both the lymph nodes and perinodal tissue can receive part of its vascularization through the branches arising in the submandibular gland and penetrating its capsule.

Given the above, the rationale during the flap harvest and especially when in the vicinity of the submandibular gland is to follow the facial artery, open the capsule of the submandibular gland and if encountered make sure that submental artery is protected. To protect level IB lymph nodes and perinodal tissue we will always include submandibular gland capsule in our flap. Sometimes it can lead to partial submandibular gland removal. To date, we have not encountered any adverse effects correlated to partial removal of the submandibular gland, such as salivary fistula or sialocele.

For a future study, we would like to examine the benefits of the preoperative mapping of lymphatic vessels that are entering the flap carrying vascularized lymph nodes using indocyanine green fluorescence. In theory, an LVA between lymphatic vessels of the flap and lymphatic vessels at the recipient site may further expedite and improve the outcome of VLNT.

## 5 CONCLUSION

A retrospective analysis of the CTA scans of the head and neck region was performed to study anatomical location, number, and size of submental lymph nodes in relation to the origin of the submental artery. It was found that the lymph nodes were randomly distributed along the mandible, both in the study population as well as within a single individual. The anatomical variation found in level IA/IB lymph nodes warrants conducting a CTA scan to maximize the number of transferred lymph nodes and aid in optimal submental flap design.

## CONFLICT OF INTERESTS

The authors declare that there are no conflict of interests.

## SYNOPSIS

Value of CTA scan in preoperative planning of submental flap as VLNT.

## Data Availability

Data available on request from the authors.
